# Development of Novel Indole-Based Bifunctional Aldose Reductase Inhibitors/Antioxidants as Promising Drugs for the Treatment of Diabetic Complications

**DOI:** 10.3390/molecules26102867

**Published:** 2021-05-12

**Authors:** Lucia Kovacikova, Marta Soltesova Prnova, Magdalena Majekova, Andrej Bohac, Cimen Karasu, Milan Stefek

**Affiliations:** 1 Institute of Experimental Pharmacology and Toxicology, CEM SAS, Dúbravská Cesta 9, 841 04 Bratislava, Slovakia; lucia.kovacikova@savba.sk (L.K.); marta.prnova@savba.sk (M.S.P.); magdalena.majekova@savba.sk (M.M.); 2 Department of Organic Chemistry, Faculty of Natural Sciences, Comenius University in Bratislava, Ilkovičova 6, 842 15 Bratislava, Slovakia; andrej.bohac@uniba.sk; 3 Biomagi, Inc., Mamateyova 26, 851 04 Bratislava, Slovakia; 4 Cellular Stress Response and Signal Transduction Research Laboratory, Department of Medical Pharmacology, Faculty of Medicine, Gazi University, Beşevler, 06500 Ankara, Turkey; karasu@gazi.edu.tr

**Keywords:** indole, pyridoindole, triazinoindole, aldose reductase, inhibitor, diabetic complications, polyol pathway, antioxidant

## Abstract

Aldose reductase (AR, ALR2), the first enzyme of the polyol pathway, is implicated in the pathophysiology of diabetic complications. Aldose reductase inhibitors (ARIs) thus present a promising therapeutic approach to treat a wide array of diabetic complications. Moreover, a therapeutic potential of ARIs in the treatment of chronic inflammation-related pathologies and several genetic metabolic disorders has been recently indicated. Substituted indoles are an interesting group of compounds with a plethora of biological activities. This article reviews a series of indole-based bifunctional aldose reductase inhibitors/antioxidants (ARIs/AOs) developed during recent years. Experimental results obtained in in vitro, ex vivo, and in vivo models of diabetic complications are presented. Structure–activity relationships with respect to carboxymethyl pharmacophore regioisomerization and core scaffold modification are discussed along with the criteria of ‘drug-likeness”. Novel promising structures of putative multifunctional ARIs/AOs are designed.

## 1. Introduction

Aldose reductase (AR, ALR2), the first enzyme of the polyol pathway, was initially connected with the onset of diabetic complications (DCs) [1,2,3,4,5,6]. More recent evidence suggests that AR also plays a key role in inflammatory pathologies and in several genetic metabolic disorders in non-diabetic subjects. In this context, aldose reductase inhibitors (ARIs) have received much attention as a promising therapeutic strategy in targeting the above-mentioned disorders [7,8,9,10,11,12,13,14,15,16,17,18,19]. 

Yet, the etiology of DCs is multifactorial since multiple mechanisms contribute to their development. Complex multifactorial etiology of DCs stems from activation of several metabolic pathways by hyperglycemia. The polyol pathway serves as a major link to other pathways responsible for glucose toxicity including the oxidative stress [20,21,22,23,24,25]. The multifactorial nature of diabetic complications represents a great challenge in the development of efficient therapy. A multi-target directed approach in prevention of diabetic complications is oriented on the rational design of chemical entities able to affect simultaneously multiple key mechanisms, since targeting just one particular mechanism may have a limited effect. This approach increases the chance of successful therapeutic intervention, decreases the risk of side effects, and is economical.

Substituted indoles are an interesting group of compounds with a plethora of biological activities. The indole scaffold, found in numerous natural and synthetic substances, is considered a useful structural subunit for drug design and discovery [26,27,28,29,30]. In our pursuit of multifactorial drugs to treat diabetic complications we were inspired by both an efficient antioxidant and free radical scavenging agent, stobadine (STB, **1a**) [25,31,32,33,34,35], a drug of hexahydropyridoindole nature, and by the highly efficient ARI lidorestat, derivative of indol-1-yl acetic acid [36]. Accordingly, both tetrahydropyridoindole scaffold evolved from stobadine, and indol-1-yl acetic acid moiety were used in our lab during the last 15 years as starting fragments in developing of several promising series of aldose reductase inhibitors/antioxidants.

**Figure 1:**
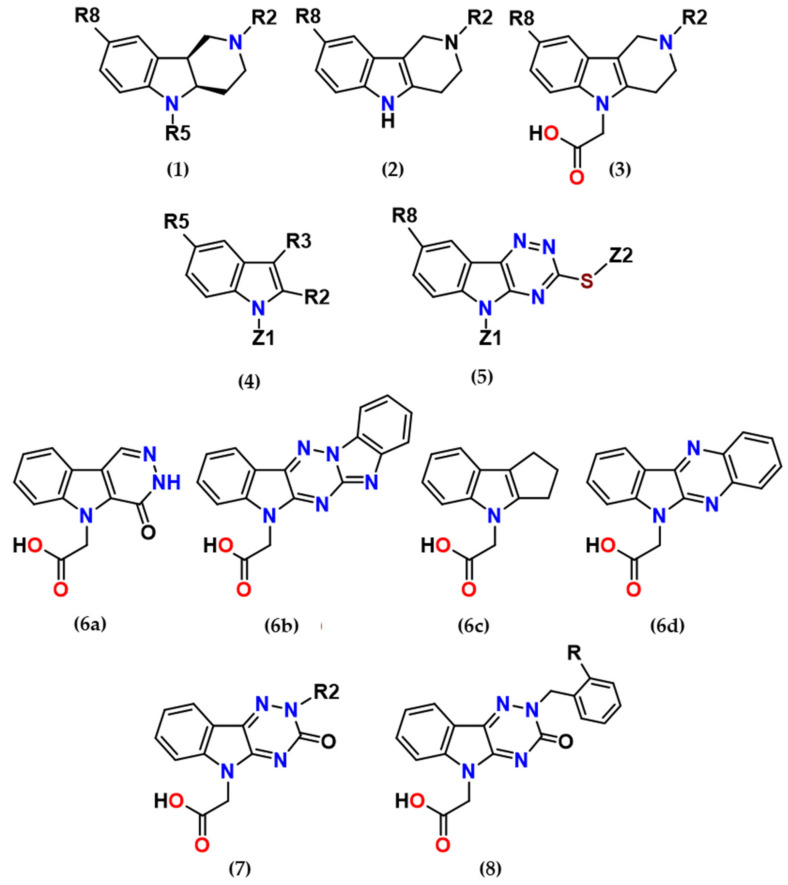
The core scaffolds of the indole-based aldose reductase inhibitors/antioxidants.

This article reviews several groups of indole-based bifunctional aldose reductase inhibitors/antioxidants designed and tested as potential drug candidates for the treatment of diabetic complications (Figure 1). 

Experimental results obtained at the level of isolated enzymes, and in the models of free radical damage in vitro are presented. The enzyme inhibition data are complemented by molecular docking simulations into the ALR2 binding site followed by results from ex vivo and in vivo models of diabetic complications. Structure–activity relationships with respect to the core indole scaffold modifications are discussed along with the criteria of ‘‘drug-likeness”. The findings thus obtained are extended to predictions of novel promising structures of multifunctional ARIs.

## 2. Studies at the Level of Isolated Enzymes and Free Radical Models in Vitro and in Silico: SAR

### 2.1. Inhibition of Aldo-Keto Reductases ALR2 and ALR1

Aldose reductase (AR, ALR2, EC 1.1.1.21) is a member of the aldo-keto reductase (AKR) superfamily, which is composed of monomeric proteins with approximately 315–330 residues, and with a molecular weight of 36 kD. It is the first enzyme of the polyol pathway and converts glucose to sorbitol in the presence of NADPH. The pathway is terminated by the second enzyme, sorbitol dehydrogenase, which converts sorbitol to fructose with NAD^+^ as a cofactor. In diabetic patients, the flux of glucose through the polyol pathway results in intracellular accumulation of sorbitol with consequent disruption of tissue osmotic homeostasis. The concurrent depletion of NADPH and the imbalance of the NADH/NAD^+^ ratio eventually lead to oxidative stress. As such, these metabolic derangements have been found implicated in the pathophysiology of diabetic complications [1,2,3,4,5,6]. Another member of the AKR superfamily is aldehyde reductase (ALR1, EC 1.1.1.2). ALR1 is present in all tissues and is responsible for the reduction of toxic aldehydes. ALR1 and ALR2 share a high degree of amino acid sequence (~65%) and structural homology and their co-inhibition may result in undesired side effects [12]. Thus, to test selectivity of novel ARIs, the closely related ALR1 is routinely employed.

Based on the premise that a bifunctional compound with joint antioxidant/aldose reductase inhibitory (AO/ARI) activities could be multifactorially beneficial, the first three series of novel derivatives (series 1 to 3), structurally based on the antioxidant drug stobadine [**1a**, (4a*R*,9b*S*)-2,8-dimethyl-2,3,4,4a,5,9*b*-hexahydro-1*H*-pyrido[4,3-b]indole] [31,32,33,34,35] were designed and synthesized. 

The parent drug stobadine (**1a**, Table 1) as an efficient reactive oxygen species (ROS) scavenger was extensively studied in multiple models of diabetic complications with the aim to attenuate the oxidative component of glucose toxicity. Indeed, under conditions of an experimental glycation model in vitro, compound **1a** was found to protect bovine serum albumin against glyco-oxidative damage [37,38,39]. Using a model of streptozotocin-diabetic rats in vivo, compound **1a** was reported to attenuate pathological changes in diabetic cardiovascular system [40,41,42,43,44], kidneys [45,46,47,48,49,50], eye lens [51,52], and retina [53], vas deferens [54], peripheral nerves [55,56], and brain [57], to decrease matrix collagen cross-linking [46,58] and to reduce plasma cholesterol [41] and triglyceride levels [41,42] in diabetic animals. Stobadine (**1a**) treatment normalized calcium homeostasis in diabetic rat heart and liver [42], produced a beneficial effect on leukocyte function [59], inhibited doxorubicin-induced apoptosis [60], and ameliorated alloxan toxicity in mice [61,62].

The rationale for designing of the novel multi-target directed/bifunctional drugs was based on an idea of endowing the stobadine scaffold bearing the antioxidant activity with a carboxymethyl functional group, a key pharmacophore of aldose reductase inhibitors. Hexahydropyridoindole stobadine (**1a**) and its tetrahydro congener **2a**, used as starting fragments of the drug design, were devoid of any ability to inhibit aldose reductase (Table 1).

**Figure 2:**
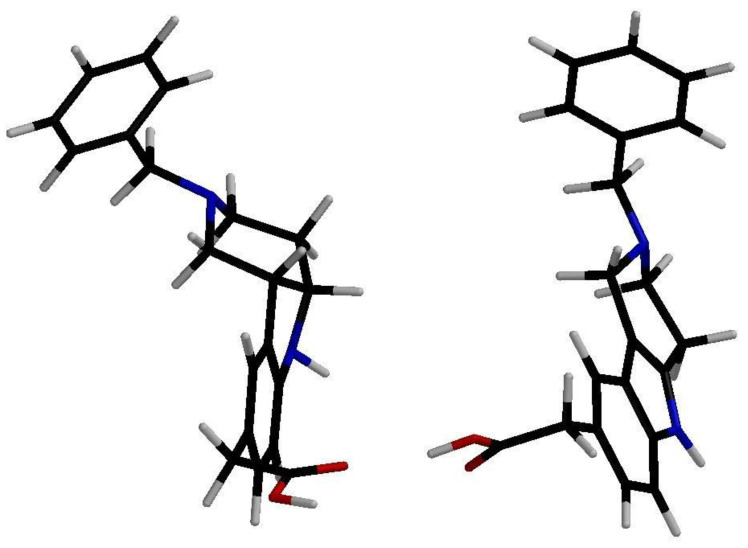
Three-dimensional (3D) structures of the low energy conformations of the hexahydropyridoindole **1c** (**left**) vs. tetrahydropyridoindole **2c** (**right**). Reprinted with permission from [63]. Copyright (2008) Elsevier.

Introduction of the carboxymethyl group into the hexahydropyridoindole **1a** at the position 8 resulted in compound **1b** with a very mild inhibition of ALR2 [**I** (%,100 µM) = 13%]. Linking the more lipophilic benzyl substituent into position 2 (compound **1c**) did not affect the inhibition efficacy significantly. Yet inhibition activities in micromolar range were recorded for the unsaturated tetrahydropyridoindole congeners **2b**–**d**. Visualization of low energy conformations of compounds **1c** and **2c** showed almost planar tricyclic moiety of the tetrahydropyridoindole **2c**, contrasting with severe space distortion of the lipophilic heterocyclic backbone of the hexahydropyridoindole **1c** (Figure 2) [63]. The presence of an extended aromatic planar region in the majority of potent ARIs is well documented as a crucial pharmacophoric element [64,65,66].

**Figure 3:**
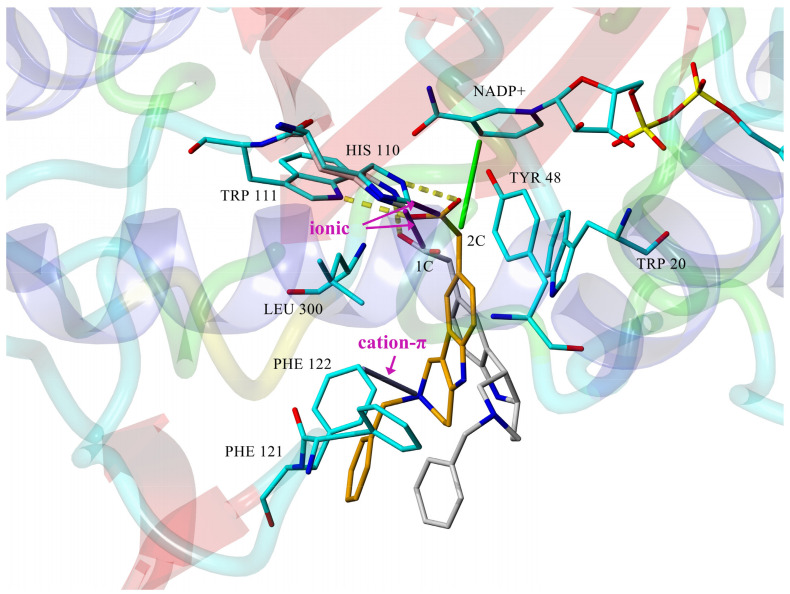
Geometry of the active site for **1c** (gray) and **2c** (mustard yellow) derivatives optimized with the whole enzyme (PDB: 1PWM). Dashed yellow lines denote the hydrogen bonds; green line outlines the hydrophobic interaction of **2c** with NADP^+^. Ionic and cation–π interactions are emphasized with magenta arrows. For transparency, only residues and NADP^+^ of **1c**-ALR2 complex are visible, with exception of His110 [63].

The computed stereoelectronic properties of compounds **1c** and **2c** along with a docking study into the ALR2-binding site (PDB: 1PWM) suggested an explanation for the higher inhibitory efficacies of the tetrahydropyridoindoles (**2**) in comparison to the hexahydropyridoindoles (**1**) shown in Figure 3. Planar conformation of the tricyclic tetrahydropyridoindole moiety of **2c** enabled closer contacts between the carboxymethyl pharmacophore and the key interaction partners His110 and NADP^+^, in comparison with sterically distorted hexahydropyridoindole structure of compound **1c** [63]. Cation–π interaction between protonated *N*2 nitrogen and benzene ring of Phe122 contributed to the stabilization of **2c**-ALR2 complex, but also prevented **2c** to adopt more advantageous position. Eliminating the basicity center at *N*2 by introducing an acyl substituent in derivative **2e** resulted in significant improvement of the inhibition efficacy [68]. The selectivity of the tetrahydropyridoindoles **2b**–**2d** in relation to ALR1 was characterized by selectivity factors (SFs) in the range of 18 to 57. It is noteworthy that compound **2c** retained ALR2 inhibitory activity even for the enzyme isolated from diabetic rats, with an IC_50_ of 16.6 μM. The selectivity index of compound **2c** even slightly increased from 18 to 21 in the enzyme preparations from diabetic rats [69]. Compound **2c** was reported [70] to be selective in relation to the enzymes of the glycolytic pathway of glucose elimination.

Shifting the carboxymethyl pharmacophore from position 8 to position 2, yielded derivatives **2f**–**h** with markedly decreased inhibition efficacy (IC_50_ >100 µM); therefore, this route of drug designing was not further followed [67].

Transferring the carboxymethyl pharmacophore from position 8 to position 5, yielded derivatives (series **3**) with markedly enhanced aldose reductase inhibition efficacy and selectivity (Table 2) [71]. Mild inhibition characterized by IC_50_ in micromolar range was recorded for compound **3a** with the isopropyl substituent in position 2. This alkylated tertiary nitrogen is characterized by a rather high basicity (pKa ~ 10, MarvinSketch Online 2016/ChemAxon), which ensures its complete protonation at physiological pH. The presence of a positive charge on the tertiary nitrogen, which predisposes these compounds to form double-charged zwitterionic species, has apparently a detrimental effect on AR inhibition efficacy. Similarly, only modest AR inhibition was recorded for structurally related zwitterionic 8-carboxymethylated pyridoindoles [63,72]. 

On the other hand, AR inhibition activity of the low basicity derivatives possessing an acyl or ethoxycarbonyl substituent on *N*2 compounds **3b**–**f** is characterized with IC_50_ values in low and medium nanomolar range (Table 2). Based on SAR in this set of compounds, the flexible carbamate moiety of compounds **3e** and **3f** appears to fit better the enzyme-binding site contrary to alkyl– and aryl-acyl– substructures of compounds **3b**–**d,** respectively. In the latter series, the inhibition efficacy decreased with increasing bulkiness of the *N*-substituent. The replacement of the methoxy group in position 8 of compound **3f** by the more polar carboxylic group in compound **3e** did not affect the resulting inhibition activity. Compounds **3b**-**f** revealed higher inhibition efficacy in comparison with the clinically used epalrestat. Compound **3b** is presently undergoing clinical evaluation under the name of setipiprant for treatment of androgenic alopecia [73]. On balance, these results establish the tetrahydropyridoindoles carboxymethylated at position 5 as a prospective scaffold for designing efficient AR inhibitors.

**Figure 4:**
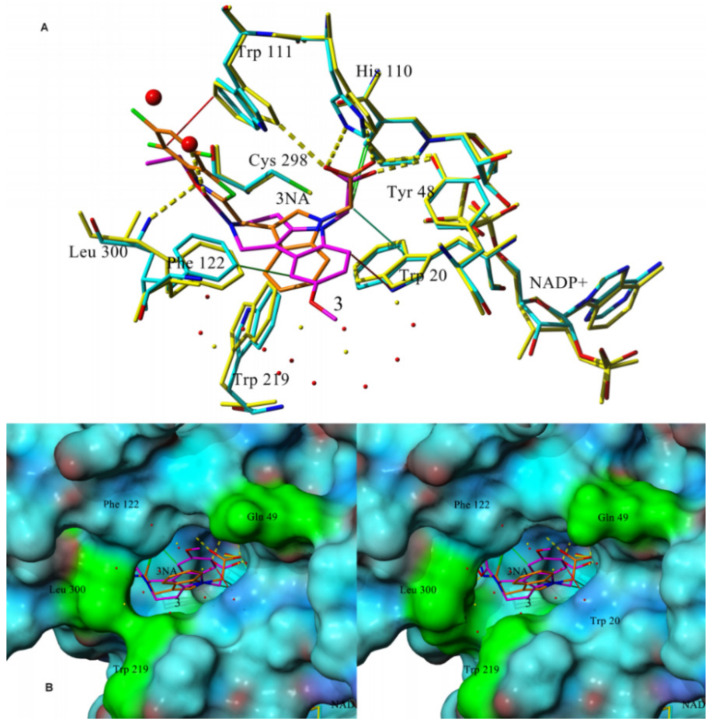
(**A**) Superposition of the binding modes of the compound **3f** (magenta) and lidorestat (orange) complexed with human AKR1B1 (PDB code 1z3n) and NADP^+^ (enzyme and NADP+ is colored by element in complex with **3f** and by yellow in complex with lidorestat). Green lines denote hydrophobic interactions, yellow dashed lines are hydrogen bonds and the red one is for π-π interaction. (**B**) Fitted molecular surface of the enzyme with superposed lidorestat (**left**) and **3f** (**right**). The surfaces are colored per atom by element type with the exception of Gln49, Trp219, and Leu300 (green). Reprinted with permission from [71]. Copyright (2017) Elsevier.

With the aim of analyzing possible interaction modes, human recombinant enzyme AKR1B1 in complex with structurally related lidorestat (PDB: 1z3n) was used for in silico docking followed by optimization of the resulting complexes in a water environment. The trial revealed several common features for the set of the most efficient inhibitors **3c**–**f**. The carboxylate group of these compounds and the one of lidorestat were found to align well. To avoid redundancy, details only for the derivative **3f** were reported. As shown in Figure 4A, the carboxylate group is directed to the main residual trio of the “anion binding pocket“, Tyr48, His110, and Trp111. 

The methylene group of the carboxymethyl substituent creates hydrophobic interactions with NADP^+^ with the exception of the least efficient inhibitor **3a**, the structure of which was, similarly as for **2c**, captured in cation–π interaction with Phe122 keeping it too far from NADP^+^. By their overall positions, compound **3f** and lidorestat take up approximately the same space in the binding site with several specific differences. The trifluorobenzothiazole part of lidorestat creates strong π–π interactions with Trp111 and Phe122, leaving no space available for water molecules in the binding pocket. However, compound **3f** binds firmly to Leu300, leaving enough space in the cavity for two water molecules and creating H-bond with one of them. Water environment plays an important role in thermodynamic balance of ligand-enzyme interactions [74]. No relevant interactions were observable for the 8-methoxy moiety, which is in agreement with the experimental findings showing no difference between the inhibition efficacies of compounds **3e** and **3f**. Compound **3f** and lidorestat induced different water arrangement near the cavity access. As shown in the fitted molecular surface (Figure 4B), lidorestat induced a narrower access to the cavity, keeping amino acids Trp219 and Leu300 closer in comparison with the complex of **3f.** The amino group of Gln49 (colored in green in Figure 4B) is perpendicular to the surface of the protein in the complex with lidorestat (Figure 4B left), while for **3f** this group is parallel to the surface (Figure 4B right) [71]. 

To test selectivity, we used the comparison to the closely related aldehyde reductase (ALR1). All compounds **3a**–**f** were found to be less active inhibitors of ALR1 compared to ALR2 (Table 2). The corresponding selectivity factors calculated for the most efficient compounds **3e** and **3f** were found to be 381 and 792, respectively. Methoxy substituent in position 8 of compound **3f** thus ensured higher selectivity than the corresponding carboxyl group in compound **3e.** This effect may be obviously assigned to the higher affinity to ALR1 of compound **3e** bearing the carboxyl group, since the alterations of the 8-metoxy vs. 8-carboxyl substituents did not affect ALR2 inhibition. In silico study revealed a strong interaction of compound **3f** with Phe122, Trp219, Leu300, and Tyr309 from the “specificity pocket”, set of residues in ALR2 which are not conserved in ALR1 [71]. The highest selectivity was recorded for compound **3d** with the corresponding selectivity factor exceeding 1000. 

One of the most efficient inhibitors, compound **3f**, was tested for inhibition of human recombinant AKR1B1 and AKR1B10, yielding IC_50_ values 84 nM and 9434 nM, respectively, pointing to high selectivity relative to AKR1B10. Zopolrestat used as reference gave for AKR1B1 the IC_50_ value of 25 nM, while for AKR1B10 it was inactive. According to the docking study, selectivity of compound **3f** towards AKR1B10 is related to the fact that in Cys298 is replaced by hydroxylated cysteine Cso299, which creates H-bond with the acetate group of compound **3f** and keeps it too far from NADP^+^ to form the hydrophobic interaction with nicotinamide ring [71]. 

**Figure 5:**
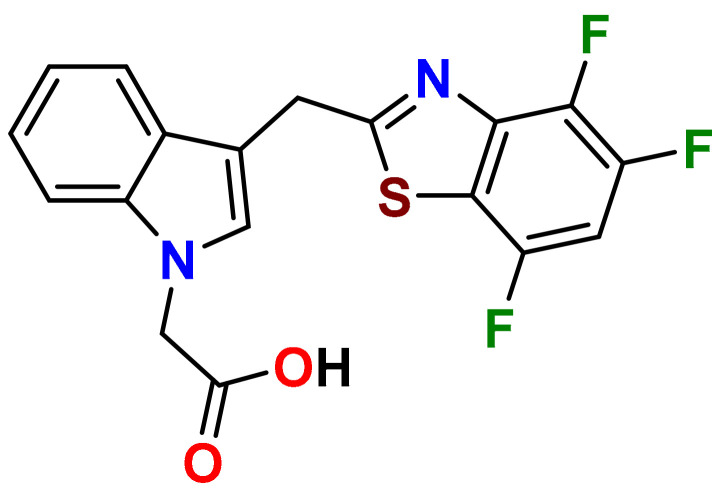
Highly efficient ARI lidorestat, a derivative of indol-1-yl acetic acid [36].

In our persistent search for highly efficient and selective ARIs, we were in the next stage of drug design inspired by lidorestat (Figure 5), one of the most efficient inhibitors of aldose reductase with reported IC_50_ value in low nanomolar region [36]. Chemically, lidorestat is a derivative of indol-1-yl acetic acid. Despite the failure of lidorestat in clinical trials and based on encouraging highly efficient derivatives **3c**–**f**, we still had believed that indol-1-yl acetic acid moiety was a promising starting fragment for drug design. Indeed, in our preliminary study [75], indol-1-yl acetic acid **4a** was identified as an ARI with a mild efficacy. We decided to employ this compound as a starting structural moiety (hit) in a fragment based drug design. By applying this approach to the virtual screening of ChemSpider database, three series (**4** to **6**) of indol-1-yl acetic acid derivatives, as summarized in Table 3, Table 4 and Table 5, were identified as promising ARIs and subjected to experimental SAR study for AR inhibition [76]. 

For comparison, two indol-1-yl propionic acid congeners (compounds **4b** and **4i**) were included in the experimental sample set. In addition, two structural variants lacking the acetic moiety on indolyl skeleton were incorporated (compounds **5b** and **5c**).

As shown in Table 3, unsubstituted indol-1-yl acetic acid (**4a**) inhibited ALR2 in low micromolar range. Insertion of a substituent at position 3 in compounds **4e**–**j**, resulted in reduced inhibitory power towards ALR2. Introduction of additional methyl substituent in position 2, did not affect the inhibition significantly, as shown in the case of compound **4f** [76].

On the other hand, combination of –CHO and –COCH_3_ substituents in position 3 with methyl in position 2 for compounds **4k** and **4l,** respectively, resulted in marked increase in inhibition efficacy as documented by substantial decrease of IC_50_ values (23- to 35-fold, respectively) in comparison with unsubstituted indol-1-yl acetic acid (**4a**) [71]. In compound **4m**, the presence of benzyloxy group in position 5 resulted in about 10-fold improvement of inhibition when compared with the unsubstituted indol-1-yl acetic acid (**4a**), based on the experimental IC_50_ values [77].

Profoundly increased inhibition was recorded for the thioxo triazine derivatives **5a** and **5g**, with IC_50_ values in submicromolar range (Table 4). Values of IC_50_ in low micromolar range were recorded for the remaining thioxotriazine derivatives **5d**–**f [76]**. The strongest inhibition of ALR2 was found for **5a** (cemtirestat, CMTI) with IC_50_ = 97 nM. The structure–activity relationship in the series of **5** and **6** points to the necessity of a concurrent presence of both, the carboxymethyl group in position 5 and the terminal sulfur to achieve best affinity. Shifting of the carboxymethyl group from indole nitrogen to sulfur in compound **5c** or its absence in compound **5b** resulted in a loss of activity. 

**Figure 6:**
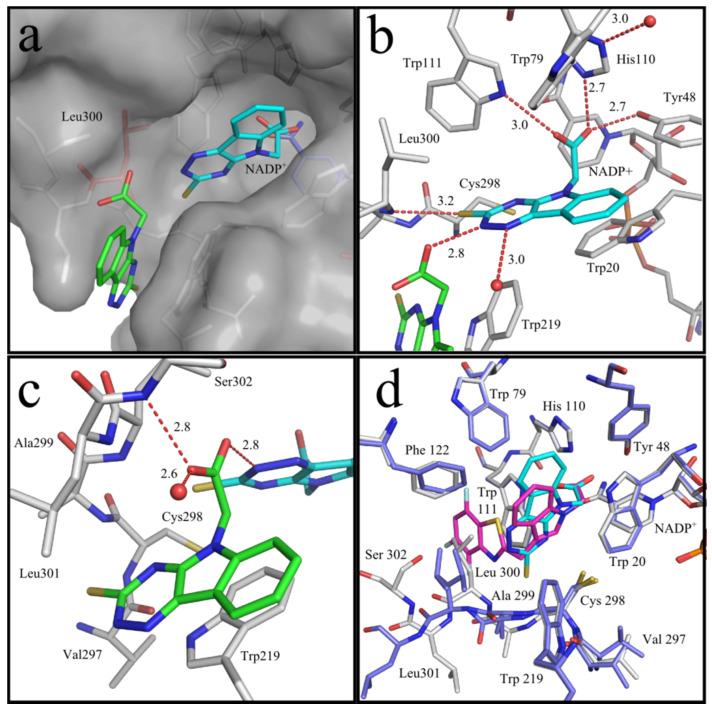
(**a**) Two molecules of compound **5a** are located in the binding site of ALR2. For NADP^+^, the first and the second molecule of compound **5a**, the nitrogen atoms are shown in blue, oxygen atoms in red, and the sulfur atoms in yellow (PDB: 4QX4). For NADP^+^, carbon atoms are shown in blue, for the first molecule of compound **5a** in cyan, and for the second molecule of compound **5a** in green. All atoms of the protein are shown in gray except Leu300, which is colored in red. Leu300 is found in a conformation that closes the specificity pocket. (**b**) Binding mode of the first molecule of compound **5a** (in cyan). Nitrogen atoms are shown in dark blue, oxygen atoms in red, and the sulfur atoms in yellow. Waters are shown as red spheres. H-bonds are indicated as red dashed lines. Atom color codes are maintained throughout the following representation. In addition to strong ion interactions of -COO^-^ anion with the protonized His110, the oxygen atoms of the carboxylate group of the inhibitor form H-bonds to Tyr48, His110, and Trp79. The sulfur atom of compound **5a** forms an H-bond to the backbone nitrogen atom of Leu300. The *N*2 of the triazine ring makes an H-bond to the second molecule of compound **5a** (in green) while the *N*1 is in contact with a water. (**c**) Binding mode of the second molecule of compound **5a**. The main interactions of the second molecule of **5a** are an H-bond between one of the carboxylate oxygen atoms to the *N*2 of the first molecule of compound **5a**, while the other oxygen atom forms H-bonds to the backbone nitrogen of Ser302 and to a water molecule. (**d**) Superposition of compound **5a** with lidorestat. The carbon atoms of lidorestat are shown in purple. The carbon atoms belonging to the protein residues of the ALR2−lidorestat complex are shown in blue. Lidorestat opens in contrast to compound **5a** the specificity pocket. Reprinted with permission from [76]. Copyright (2015) American Chemical Society.

High resolution X-ray crystallography of the human recombinant AKR1B1 enzyme complexed with cemtirestat (**5a**, PDB: 4QX4) demonstrated a unique pattern of cemtirestat binding, with the specificity pocket closed, contrary to the interaction of the structurally related lidorestat [76]. As shown in greater detail in Figure 6a, two molecules of the inhibitor **5a** were visible in the determined difference electron density, one in the entrance of the binding pocket and the second one as expected inside the binding pocket. The oxygen atom of the carboxylate group of the first inhibitor molecule **5a** and *N*2 of the second one form an H-bond (2.8 Å, Figure 6b). The proton is most likely contributed by the nitrogen of the triazine ring of the second molecule of compound **5a**, as we assume that the carboxylate was deprotonated under the applied conditions. The other carboxylate oxygen atom of the first molecule of compound **5a** is in H-bond distance to the backbone nitrogen of Ser302 (2.8 Å). Furthermore, the aromatic core skeleton makes a face-to-face π-stacking to Trp219 (3.4 Å, Figure 6c). Figure 6d depicts the superposition of compound **5a** with lidorestat in the binding pocket of AKR1B1. In contrast to compound **5a**, lidorestat opens the specificity pocket. Molecular dynamics simulations corroborated [78] the above results of the X-ray crystallographic assay.

The other polycyclic derivatives **6a**–**d** revealed moderate inhibition of ALR2 characterized by IC_50_ values in low micromolar range (Table 5).

All tested compounds of the series **4** to **6** were found to be less active on ALR1 compared to ALR2. It should be noted that the most selective compound **5a**, with a selectivity factor above 400, is also the most potent ARI in the series **4** to **6** (Table 3, Table 4 and Table 5).

Considering excellent “lead-likeness” of cemtirestat (**5a**) [76], we proceeded with optimization of its thioxotriazinoindole scaffold by replacing sulfur with oxygen, with the aim to improve the inhibitory efficacy and selectivity. Based on preliminary molecular modeling and docking calculations, a series of 2-(3-oxo-2*H*-[1,2,4]triazino[5,6-*b*]indol-5(3*H*)-yl)acetic acid derivatives **7a**–**d** was proposed, synthesized and their AR inhibitory efficacy and selectivity determined [79]. 

The preliminary molecular modeling and docking study on protein conformer from PDB: 4QX4 pointed to preferable fitting of the O-derivative into the inhibitor-binding site of AR. Indeed, the O-variant of cemtirestat (**5a**), oxotriazinoindole, compound **7a** (OTI), demonstrated significantly increased inhibition efficacy characterized by IC_50_ values 2-3 times lower compared to compound **5a**, depending on the solvent used. SAR evaluation in the series of novel OTI derivatives revealed that the presence of simple substituents at *N*2 position of compound **7a** decreased their ALR2 inhibition efficacy. The most remarkable decrease was recorded for compound **7c** with the lipophilic benzyl substituent.

All compounds evaluated were less efficient inhibitors of ALR1 compared to ALR2. With exception of compound **7b**, the ALR1 IC_50_ values of the OTI derivatives (**7a**, **7c,** and **7d**) were found to be over 100 µM. For these derivatives, the percentage inhibition I(%) at 100 µM concentration of the inhibitor was determined. Estimates of the particular selectivity factors calculated for the most efficient inhibitors **7a** and **7d** were found to be >2381 and >1177, respectively. It is striking to observe the enormous increase of the selectivity factor in the couple cemtirestat (**5a**) vs. its O isostere **7a** (from 422 to >2381). Molecular docking into the binding site of ALR1 offered a feasible explanation: cation–π interaction of protonated Arg312 in ALR1 with the aromatic ring of O-derivative **7a** supported by two H-bonds was found to keep this molecule out of reach of NADP^+^ cofactor in contrast to more tightly attached cemtirestat **5a** (Figure S52 in [79]). In addition, a hydrophobic interaction between the methylene residue of an acetate group of compound **7a** and **5a,** respectively, and the nicotinamide ring of NADP^+^ should also be taken into consideration [74]. Based on the IC_50_ values shown in Table 6, compound **7a** inhibits ALR2 more efficiently (IC_50_ = 42 nM) than the reference inhibitor epalrestat (IC_50_ = 227 nM, Table 2). 

**Figure 7:**
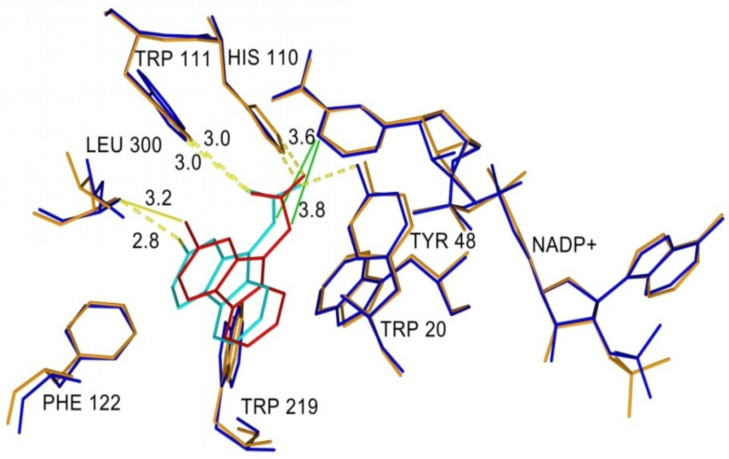
Superposition of compound **7a** and cemtirestat (**5a**) in the binding site of AKR1B1 (PDB: 4QX4). Cemtirestat (**5a**) is colored in red and the corresponding amino acid residues in orange. Compound **7a** is shown in cyan and the complementary amino acid residues in dark blue. H-bonds are indicated as yellow and hydrophobic interactions as green lines. Reprinted with permission from [79]. Copyright (2020) American Chemical Society.

Figure 7 shows superposition of cemtirestat (**5a**) and its O-analogue **7a** in the inhibitor-binding site of AKR1B1. Polar carboxymethyl groups of both compounds were found to align well. Slight distortion of the fused planar aromatic system was apparently caused by higher rotational flexibility of the carboxymethyl moiety of compound **7a** (in cyan) owing to less bulky oxygen compared to the sulfur of the original thioxotriazinoindole **5a** (in red, van der Waals radius of O is 1.4 Å, while for S it is 1.9 Å). As a result, compound 7a was able to create stronger H-bond with Leu300 (contact distance 2.8 Å vs. 3.2 Å) and gave more favorable hydrophobic interaction (2.0 kJ/mol for compound **7a** vs. 0.6 kJ/mol for cemtirestat **5a**) with NADP+ (contact distance 3.6 Å vs. 3.8 Å). Compound **7a** interacts with the residues of the specificity pocket Phe122 and Leu300 while letting the specificity pocket closed, which is in line with the previously published crystal structure of AKR1B1 complexed with cemtirestat (**5a**, PDB: 4QX4). These in silico findings provided an explanation of better fitting of the O-derivative **7a** into the binding site of AR when compared with its parent molecule of cemtirestat (**5a**).

In the next stage, we focused on the development of novel oxotriazinoindole inhibitors of aldose reductase designed to fit an unoccupied ALR2 pocket shown in PDB: 4QX4 surrounded by the amino acid residues Trp219, Ala299, and Leu301. In order to utilize this pocket for additional ligand–enzyme interactions, novel *N*-benzyl(oxotriazinoindole) derivatives **8a**–**d** have been designed and developed (Table 7) [80].

Molecules **8a**–**d** were tested for their ability to inhibit the reduction of *D,L-*glyceraldehyde using ALR2 isolated from the rat eye lenses. Unsubstituted benzyl analogue **7c** was used as a reference inhibitor. To assess selectivity, we used a structurally related detoxification enzyme (an antitarget), aldehyde reductase (ALR1), isolated from the rat kidneys. We found out that all substituted *N*-(benzyl) derivatives **8a**–**d** exhibited from 2- to 6-fold better inhibitory efficacy than unsubstituted analogue **7c**. In addition, they also revealed low inhibition of ALR1, which resulted in good (compounds **8b**–**d**) and excellent (compound **8a**) enzyme selectivity (Table 7). 

**Figure 8:**
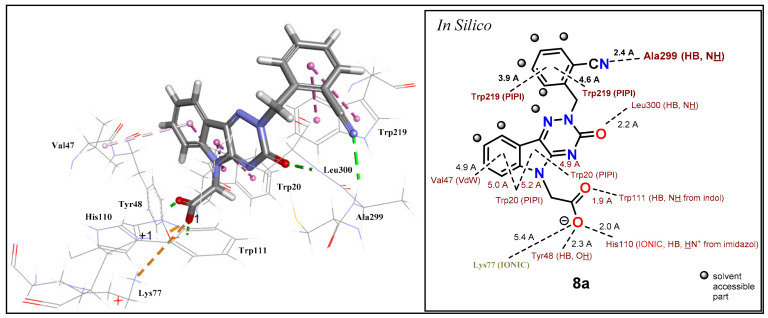
Predicted binding pose and interaction analysis of **8a** in the binding site of an ALR2 (PDB: 4QX4). Reprinted with permission from [80]. Copyright (2021) Elsevier.

The results confirmed the proposed additional interactions of substituted *N*-(benzyl) derivatives **8a**–**d** within the interactive pocket of ALR2. The best ALR2 inhibition (IC_50_ = 76 nM) and selectivity relative to ALR1 (SF = 1316) was obtained for compound **8a** containing a *N*-(2-cyanobenzyl) group. Predicted binding position of compound **8a** in an active site of ALR2 shows an H-bond of a cyano group with Ala299 from backbone (2.4 Å). Moreover, the aromatic ring of a benzyl moiety forms two π–π interactions with Trp219 (3.9 and 4.6 Å) (Figure 8). 

In spite of the predicted similar positions and interactions of compounds **8d** and **8a**, the derivative **8d** exhibited about 3-times lower inhibitory activity characterized with IC_50_ = 244 nM. The additional reason for the lower activity of compound **8d** could be the desolvation and the rotation penalty caused by a –CH_2_OH group. Desolvation penalty effect is a subject of recent publication [81].

**Figure 9:**
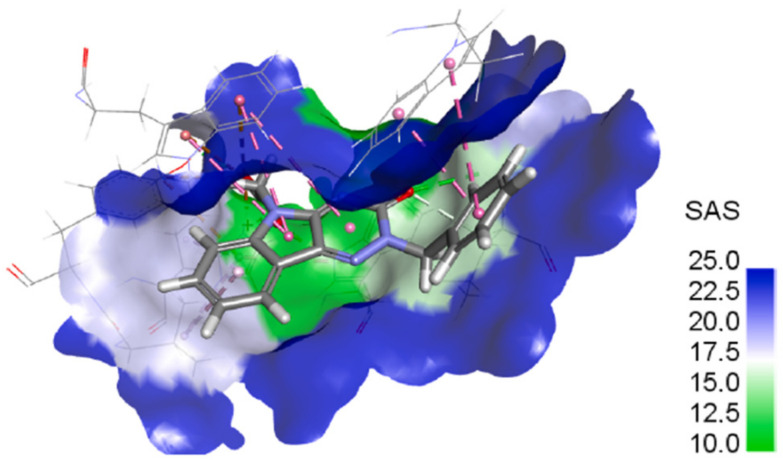
Solvents accessible surface of the unsubstituted benzyl derivative **7c**. Blue color indicates water accessible parts. Reprinted with permission from [80]. Copyright (2021) Elsevier.

Derivatives **8b,c** (–CONH_2_, –COOH, respectively) revealed 2- to 3-fold lower inhibitory activity in comparison to compound **8a** (–CN). Besides desolvation penalty, the lower activity of a carbamoyl derivative **8b** could be also caused by conformational penalty of –CONH_2_ group. The symmetry of the delocalized carboxylate group in **8c** (–COO^-^) exhibited better inhibition activity than –CONH_2_ in compound **8b** and –CH_2_OH in compound **8d**. In addition, the outer part of the studied binding pocket and the benzyl group are well water accessible (Figure 9) and, as a consequence, the benzyl group could provide two conformers. 

The first conformer allows the formation of the predicted H-bond with an orientation of a polar group inside the pocket and the second conformer prefers an orientation towards the solvent. Consequently, very polar groups would not be oriented inside the pocket, where the predicted H-bonds could be formed, but owing to solvation, they would remain oriented towards a water environment out of the pocket. Therefore, significant H-bond with Ala299 or Leu301 would not be formed as predicted and thus the least solvated derivative **8a** (–CN) revealed the highest inhibitory activity.

Table 8 summarizes the human recombinant enzyme AKR1B1 inhibition data of the most promising ARIs representing the four leading scaffolds. The inhibition efficacies and selectivities of AKR1B1 are compared with those of rat lens ALR2. 

### 2.2. Antioxidant Activity

**Figure 10:**
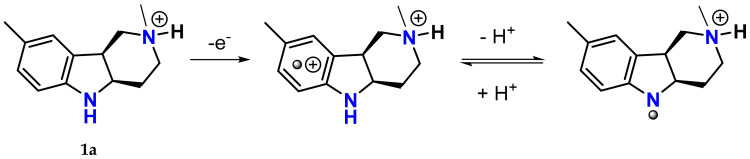
One-electron oxidation of protonated stobadine (**1a**) in a water solution followed by deprotonation of the indole nitrogen of its oxidized form to give a resonance-stabilized, nitrogen-centered radical. Modified according to [82].

As mentioned above, the hexahydropyridoindole scaffold of stobadine (**1a**) and its tetrahydro-congener **2a** had been used as core structures in designing bifunctional agents combining antioxidant activity with aldose reductase inhibitory action. The antioxidant and free radical scavenging ability of stobadine stems from ability of the indole to form a resonance-stabilized nitrogen-centered radical, which is formed after one-electron removal followed by deprotonation (Figure 10) [82]. It has been well documented that structural alterations in the close proximity of the indole nitrogen, affecting its hydrogen donating ability, are crucial for the free radical scavenging efficiency [83,84].

Aromatization of the hexahydropyridoindole skeleton of stobadine (**1a**) into its tetrahydro-congener **2a** significantly lowered the antioxidant activity, while acetylation of the indole nitrogen in compound **1e** completely abolished the ability to scavenge free radicals [83]. The results are fully in agreement with the notion that the antioxidant activity of stobadine and the related pyridoindoles is mediated via the indole nitrogen center [82,84,85].

As shown in Table 9, in agreement with our previously published results [83,84], stobadine (**1a**) and its carboxymethylated hexahydropyridoindole derivatives **1b** and **1c** rapidly reacted with DPPH, their antiradical activities being from 4 to 8 times higher than those of their tetrahydropyridoindole analogues **2a**–**d** in which nitrogen lone pair is involved in the indole aromatic system. In the series of hexahydropyridoindoles **1a**–**c**, the presence of the carboxymethyl group in position 8, decreased the DPPH scavenging ability markedly. On the other hand, analogous carboxymethyl substitution in the tetrahydro- congeners **2a**–**d** did not affect the antioxidant activity significantly. Notably, substitution of the methyl group in position 8 by the more electron-donating methoxy substituent yielding compound **1d,** with augmented electron density on the aromatic ring, markedly increased the free radical scavenging activity compared to the parent stobadine (**1a**). The initial rate of DPPH decolorization by compound **1d** was comparable with that of equimolar trolox. The presence of the carbamate moiety in position 2 decreased the basicity of the nitrogen, without affecting the intrinsic chemical reactivity with free radicals [86]. However, it had profound consequences for the bioavailability of the drug as discussed below (Section 2.3). Acetylation of the indole nitrogen (position 5) of the parent stobadine (**1a**) yielded a completely non-active derivative **1e** (Table 9). This finding is in the line with the above-mentioned mechanism of free radical scavenging action of the hexahyropyridoindoles according to which the presence of a free hydrogen on the indole nitrogen (position 5) is a prerequisite for the antiradical activity. 

Introducing the carboxymethyl pharmacophore into indole nitrogen (position N5), yielded derivatives **3a**–**f** with appreciable aldose reductase inhibition efficacy as shown in Table 2, yet with abolished antioxidant activity as reported in [67,71].

Similarly, in the series **4**, indol-1-yl acetic acid (**4a**) was devoid of any antioxidant activity as shown in Table 10. On the other hand, in the case of the indol-3-yl acetic acid derivatives with unsubstituted nitrogen **4c**, **4d**, the antiradical activity higher than that of the structurally related reference antioxidant melatonin was recorded. The electron donor substituent -OCH_3_ in the more efficient derivative **4d** facilitates the delocalization of the unpaired electron of the intermediate indolyl radical originating after free radical encounter [75]. 

In the series **5**, the antiradical activity of the most efficient AR inhibitor cemtirestat (**5a**) markedly exceeded that of equimolar melatonin (Table 10). On placing an isopropyl group on the S atom resulting in compound **5f**, thus preventing thione–thiol tautomerism and deprotonation of the –SH group, the antiradical activity of compound **5f** was completely lost [87].

**Figure 11:**
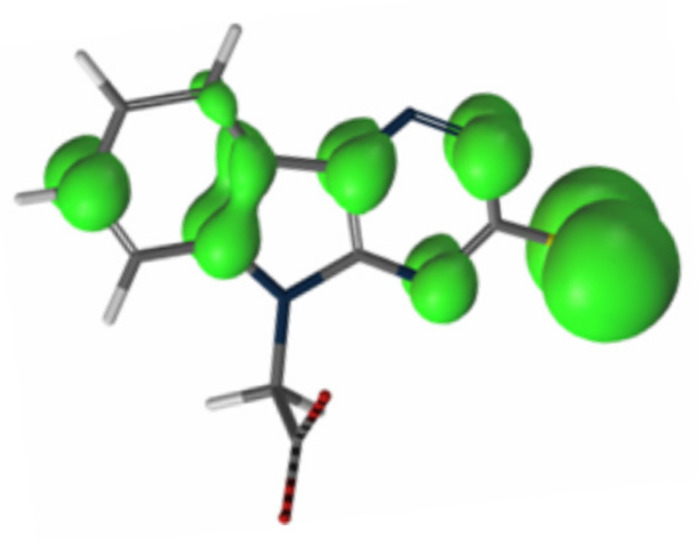
Spin density of one-electron-oxidized radical of cemtirestat (**5a**) [88].

Taking into account the above findings and spin density calculations, an S-centered charge localization in one electron-oxidized radical of cemtirestat (**5a**) was suggested as shown in Figure 11 [88]. The total stoichiometry of DPPH scavenging by cemtirestat (**5a**) was determined. Under the experimental conditions used, one molecule of compound **5a** was found to quench about 1.5 DPPH radicals [87].

As shown in Table 10, the oxygen isosteric congeners **7a** and **7d** of cemtirestat (**5a**) affected the kinetics of DPPH decolorization only marginally, pointing to their decreased free radical scavenging ability compared to that of sulfur- containing cemtirestat (**5a**). Yet the free radical scavenging efficacy of compounds **7a** and **7d** still exceeded that of the equimolar melatonin used as a standard. 

**Figure 12:**
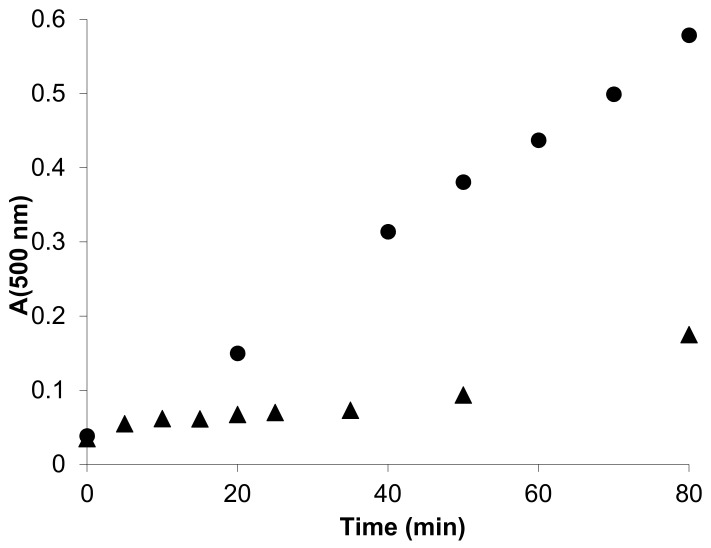
Typical kinetic curve of AAPH-induced peroxidation of DOPC liposomes (●) and the appearance of inhibition period in the presence of compound **2c** (250 µM, ▲). DOPC liposomes (0.8 mM) were incubated in the presence of AAPH (10 mM) in phosphate buffer (pH 7.4, 20 mM) at 50 °C. Reprinted with permission from [63]. Copyright (2008) Elsevier.

Unilamellar 1,2-dioleoyl-sn-glycero-3-phosphocholine (DOPC) liposomes were used as model membranes to study an overall antioxidant activity. Peroxidation of liposomes was induced by the water-soluble radical generator 2,2′-azobis(2-methylpropionamidine) dihydrochloride (AAPH) which simulates an attack by free radicals from the aqueous region. The carboxymethylated tetrahydropyridoindole **2c** effectively suppressed oxidation and gave a distinct lag phase in the lipid peroxide accumulation curve (Figure 12). As shown in Table 11, the overall antioxidant efficiency, based on IC_50_ values, of the carboxymethylated congener **2c**, is significantly lower in comparison with the parent compound **2a** in spite of the fact that the intrinsic antiradical activity determined in the DPPH test is similar for both compounds (Table 9). The lipophilicity drop caused by the presence of the polar carboxymethyl group in **2c** is obviously responsible for the marked decrease in the overall antioxidant efficacy in liposomes. A similar situation was recorded for the hexahydropyridoindole congeners **1a** vs. **1c**.

In the homogeneous model system of DPPH in ethanol, the antioxidant activity of a compound is determined by its intrinsic chemical reactivity with the radicals. In membranes, however, the apparent reactivity may be different since it is affected by the distribution ratio of the antioxidant between water and lipid compartments [63].

### 2.3. Physicochemical Properties and Bioavailability

The bioavailability of the majority of drugs depends only on their lipophilicity. The more polar the drug, the less efficiently it can penetrate the lipid membrane. Increasing the lipophilicity improves the absorption. Extremely lipophilic compounds have disadvantage of poor solubility in water and very slow absorption. 

Obviously, when considering biological availability of a drug in general, the molecule has not only to be lipophilic enough, as characterized by corresponding distribution coefficient (logP), but also neutral at the actual pH, the property that is affected by acidobasic behavior. For acidic and basic drugs, pKa constants markedly affect distribution between water and lipid compartments. In the case of highly lipophilic drugs, it is advantageous if their pKa is not too far away from the pH neutral point. In their ionized form, they are water soluble, while in their neutral form, with which they are in equilibrium, they are lipophilic and membrane penetrable. In the context of the compounds reviewed here, this notion can be illustrated by the two following examples.

#### 2.3.1. Example 1. Hexahydropyridoindole Antioxidants

**Figure 13:**
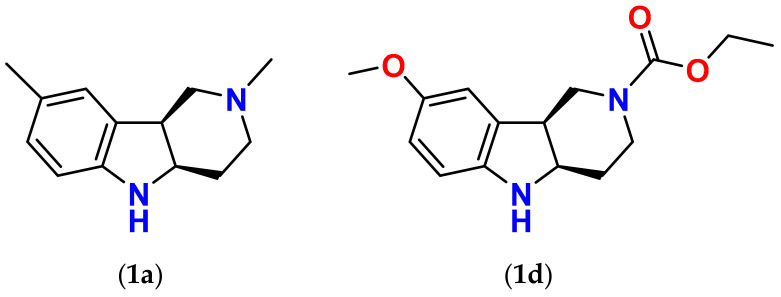
Structural alterations of the hexahydropyridoindole scaffold affect both antioxidant activity and bioavailability as exemplified by compounds **1a** and **1d**.

The substituted hexahydropyridoindoles **1a** and **1d** (Figure 13) have been postulated as chain-breaking antioxidants [62,82,83,84,85,86,90,91]. The center of the antioxidant activity was identified to reside at the indole nitrogen. Structural alterations in the close proximity of the indole nitrogen, especially aromatic substitution in positions *o-* and *p-*, were found to influence the antioxidant efficacy. On the other hand, alteration in the synthetically accessible position *N*2 provides an opportunity to vary basicity and lipophilicity of the compounds, thus modifying bioavailability, without affecting the intrinsic reactivity with free radicals [83,84].

The most efficient antioxidant of the hexahydropyridoindole series **1d** was projected with the aim to increase the free radical scavenging activity compared to the parent stobadine (**1a**) by substituting the aromatic methyl group with the more electron-donating methoxy group. At the same time, for the sake of bioavailability improvement, the basicity decrease of the molecule was achieved by replacement of the methyl substituent in position *N*2 by an appropriate acyl substituent, designed so that the lipophilicity of the parent molecule **1a** would not change significantly.

Based on the DPPH test, the free radical scavenging activity of compound **1d** was more than twice higher than that of equimolar stobadine (**1a**) and comparable with that of the standard trolox (Table 9). In this homogeneous cell-free system, antioxidant activity reflects the intrinsic chemical reactivity towards radicals. In membranes, however, the relative reactivity of antioxidants may be different since it is determined also by additional factors such as mutual location of the antioxidant and radicals at the membrane, ruled predominantly by their actual distribution ratios between water and lipid compartments.

Based on partition coefficients, compounds **1d** and **1a** have very similar lipophilicity, characterized by the respective calculated partition coefficients (ClogP) values of 1.95 and 1.79 [86]. At pH 7.4, however, with regard to the variance of basicity of the proton-binding center represented by the piperidine (*N*2) nitrogen, their actual calculated distribution ratios (ClogD) differ markedly. The indole nitrogen (N5) with very low pKa (for compound **1a** pKa_1_ ~ 3), remains unprotonated at pH 7.4. For compound **1a**, as a high basicity N-methyl derivative with pKa_2_ = 8.5 [86], the acidobasic equilibrium with respect to the piperidine nitrogen (*N*2) is strongly shifted to its protonation at neutral pH, which is reflected by the low distribution ratio ClogD regardless of the high partition coefficient ClogP. In contrast, in compound **1d**, the acyl substituent at *N*2 lowers the basicity of this site profoundly (pKa_2_= −3.7). Therefore, the protonation of this nitrogen is negligible at physiological pH, which is reflected by high actual distribution ratio ClogD at pH 7.4, reaching almost the value of the partition coefficient ClogP [86]. 

Table 12 compares the antioxidant effects of the hexahydropyridoindoles **1a** and **1d** in the cellular system of isolated rat erythrocytes oxidatively stressed by AAPH- and t-BuOOH-derived peroxyl radicals, respectively. In the first case, when the red blood cells were exposed to the peroxyl radicals generated in the medium outside the cells by thermal decomposition of hydrophilic AAPH initiator, compound **1d** was found to be less protective than equimolar stobadine (**1a**) at 10 μM concentration. On the other hand, when peroxyl radicals were generated inside the erythrocytes, by degradation of the lipophilic t-BuOOH, compound **1d** protected the cells more efficiently than equimolar stobadine (**1a**) at 10 μM concentration. To account for the apparent discrepancy, the variance of basicity of compound **1a** vs. compound **1d** should be taken into consideration as indicated above. 

#### 2.3.2. Example 2: ARIs of Zwitterionic Nature

**Figure 14:**
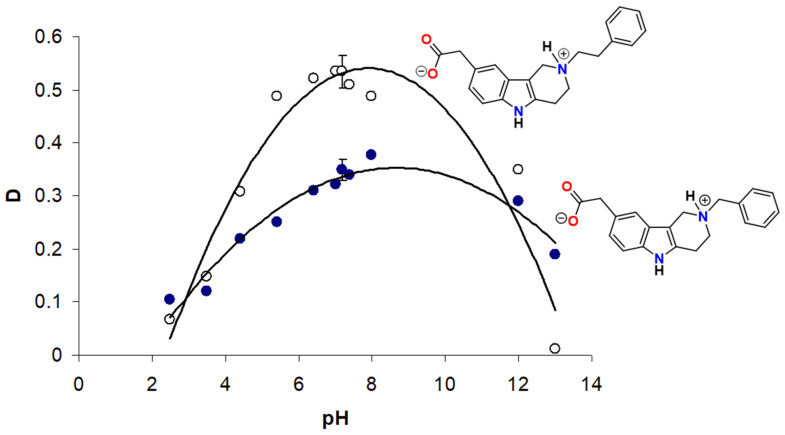
pH-distribution profile of the zwitterionic compounds **2c** (●) and **2d** (○) in 1-octanol/buffer system. D, experimentally determined distribution ratios at a given pH. Reprinted with permission from [63]. Copyright (2008) Elsevier.

The presence of a basicity center at the tertiary nitrogen in compounds **2b**–**d**, in addition to the acidic carboxylic group, predisposes these compounds to form double charged zwitterionic species. The pH-lipophilicity profile experimentally determined for compounds **2c** and **2d** in the system of 1-octanol/buffer was characterized by a bell-shaped curve, with a maximal distribution ratio near neutral pH (Figure 14) [63]. The presence of zwitterions was experimentally proved [72].

The isoelectric pH lying closely to the physiologically relevant pH 7.4 predisposes these compounds for good bioavailability. This behavior is in contrast with that of the acidic ARIs (not possessing a basic group) whose carboxylic acid function is ionized at neutral pH resulting in a sharp drop in distribution ratios and poor biological availability under physiological conditions.

## 3. Biological Activity in the Rat Models of Oxidative Stress and Diabetic Complications 

### 3.1. Antioxidant Activity of Cemtirestat (**5a**) in DOPC Liposomes Oxidatively Stressed by AAPH

**Figure 15:**
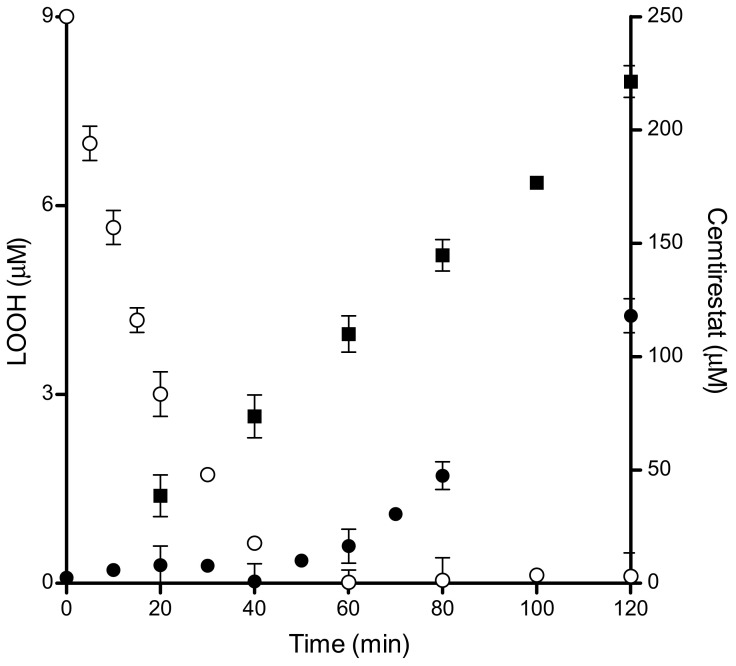
Time-dependent peroxidation of DOPC liposomes induced by AAPH in the absence (■) and in the presence of compound **5a** (250 µM, ●) and **5a** degradation curve (○). DOPC liposomes (0.8 mM) were incubated in the presence of AAPH (10 mM) in phosphate buffer (pH 7.4; 20 mM) at 50 °C. Reprinted with permission from [89]. Copyright (2020) Elsevier.

A simple membrane model of unilamellar DOPC (1,2-dioleoyl-sn-glycero-3-phosphocholine) liposomes was employed to assess the antioxidant action of the aldose reductase inhibitor **5a** (cemtirestat) in comparison with that of the standard melatonin and trolox. The hydrophilic azo-initiator AAPH [2,2′-azobis(2-methylpropionamidine) dihydrochloride] was used to generate peroxyl radicals in water phase. Compound **5a** efficiently hindered the peroxidation and produced a distinct initial lag phase of about 40 min (Figure 15). Spectrophotometric assay revealed significant consumption of compound **5a** during the induction period. After total depletion of compound **5a**, lipid peroxidation resumed with the same rate as that in the absence of the drug [89]. Interestingly, compound **5a** blocked the peroxidation even when supplemented to pre-peroxidized liposomes in the stage of the advanced peroxidation. This finding can be explained by a direct interference of compound **5a** with the chain propagation within the liposomes. 

Based on the IC_50_ values obtained, compound **5a** inhibited liposome peroxidation slightly more efficiently than melatonin, yet less effectively than trolox (Table 11) [89]. 

### 3.2. Antioxidant Activity of Cemtirestat (**5a**) in the ex Vivo Model of t-BuOOH Induced Hemolysis 

**Figure 16:**
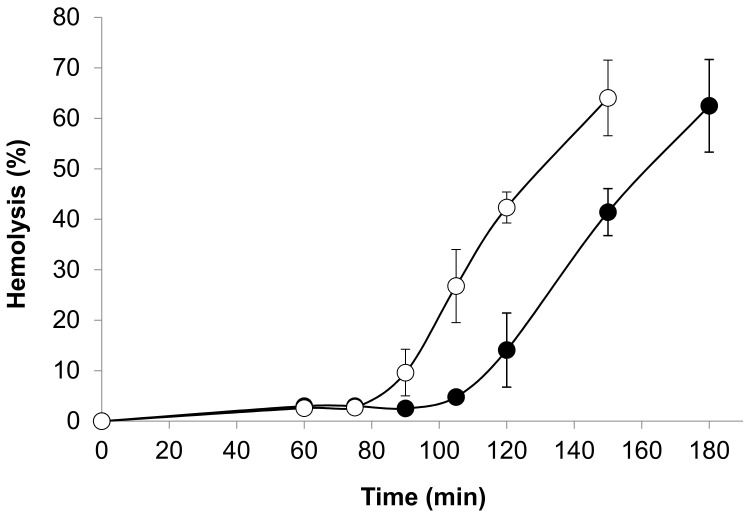
Hemolysis curves induced by *t*-BuOOH. Erythrocyte suspensions (1.5%) were incubated with 250 μM *t*-BuOOH in the presence of 100 μM (-●-) of **5a** (cemtirestat). Control incubations (-o-). Modified according to [87].

Red blood cells have been used as a model to investigate oxidative damage in biomembranes. Exposure of erythrocytes to free radicals may lead to a number of membrane changes resulting eventually in hemolysis. Lipid peroxidation and protein oxidation are likely to play a key role in the hemolytic process. In this model, oxidative damage of plasma membrane was induced by *t*-BuOOH. Owing to its high lipophilicity, *t*-BuOOH is rapidly taken up by red blood cells, which is followed by generation of peroxyl radicals [92]. Plasma membrane attack by reactive oxygen species from the intracellular region is simulated in this model. As shown in Figure 16, rat erythrocytes exposed to 250 μM *t*-BuOOH underwent progressive hemolysis followed by the release of hemoglobin. The onset of *t*-BuOOH-induced hemolysis was shifted from the starting zero point by the time interval designated as lag period. In the presence of compound **5a**, the lag period increased significantly in a concentration-dependent manner. At 1 mM concentration of compound **5a**, no hemolysis occurred during the 3-h incubation period [87]. Based on lag phase prolongation, it can be deduced that the erythrocytes were protected by compound **5a** against *t*-BuOOH-induced hemolysis. Considering the fact that the damaging oxygen species are generated from *t*-BuOOH intracellularly, the above finding indicates that compound **5a** is taken up readily by the cells.

### 3.3. Inhibitory Effect of ARIs on Sorbitol Accumulation in Isolated Rat Eye Lenses ex Vivo

Table 13 summarizes the effects of the aldose reductase inhibitors **2c**, **3d**, **3f**, **4m**, **5a**, and **7a** on sorbitol accumulation in the isolated rat eye lenses incubated ex vivo with high glucose in comparison with standard epalrestat. There were recorded concentration-dependent inhibitory effects on the basis of which the IC_50_ values were extrapolated.

The highest efficacy with estimated IC_50_ values around 10 µM was shown for the most efficient ARIs **3d**, **3f**, and **7a**. The above findings indicate uptake of the compounds studied by the eye lens tissue followed by their interference with the polyol pathway via inhibition of the cytosolic ALR2. Interestingly, the organ inhibition efficacy of **2c** is comparable with that of the markedly more efficient ARIs **4m** and **5a**, which likely could be explained by higher bioavailability owing to the zwitterionic nature of compound **2c**.

### 3.4. Inhibitory Effect of ARIs on Sorbitol Accumulation in the Sciatic Nerve of STZ Diabetic Rats In Vivo

In untreated diabetic rats, significant elevation of sorbitol concentration in the sciatic nerve was recorded. The compounds studied were administered intragastrically (50 mg/kg/day) for five consecutive days. The treatment resulted in about 20% inhibition of sorbitol accumulation in the sciatic nerve as summarized for compounds **2c, 3f,** and **5a** in Table 14. 

This result points to a ready uptake of the compounds into the central compartment after their intragastrical administration followed by their supply to the peripheral nerves and inhibition of AR-mediated sorbitol accumulation. Yet, the sorbitol levels in the nerves were not normalized to control values. Optimization of the dosage regimen may improve the therapeutic outcome.

## 4. Conclusions and Future Perspectives

With regard to the multifactorial pathophysiological origin of diabetic complications, a therapeutic approach based on the use of multi-target directed drugs has been forwarded. Based on the premise that a bifunctional compound with joint antioxidant/aldose reductase inhibitory (AO/ARI) activities could be multifactorially beneficial, we were inspired by both an efficient antioxidant stobadine (**1a**), a drug of hexahydropyridoindole nature, and by the highly efficient ARI lidorestat, derivative of indol-1-yl acetic acid. Stobadine (**1a**) as an efficient ROS scavenger was extensively studied in multiple models of diabetic complication with the aim to attenuate the oxidative component of glucose toxicity. Lidorestat belongs to a broad class of acidic aldose reductase inhibitors with characteristic carboxymethyl pharmacophore.

**Figure 17:**
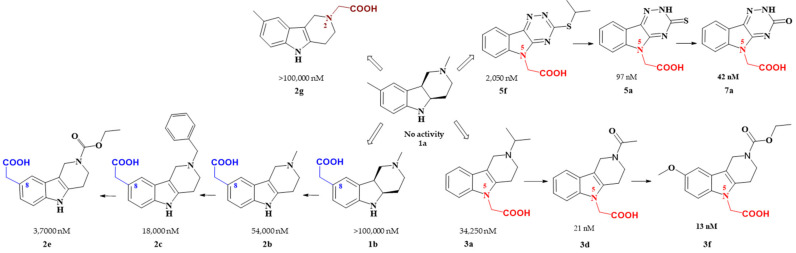
Carboxymethyl pharmacophore regioisomerization in ALR2 inhibitory activity optimization starting from the non-active hexahydropyridoindole scaffold **1a**.

At the very beginning of the drug design, we employed the hexahydropyridoindole moiety of stobadine (**1a**) as a starting scaffold, which was sequentially endowed with the carboxymethyl pharmacophore in three different synthetically accessible positions (2, 5 and 8). SAR study of the novel derivatives in relation to the carboxymethyl pharmacophore regioisomerization and core scaffold modification resulted in developing of several promising series of aldose reductase inhibitors/antioxidants as summarized in Figure 17.

Starting from the efficient hexahydropyridoindole antioxidant stobadine (**1a**), two series of hexahydro- and tetrahydropyridoindoles carboxymethylated in position 8 were synthesized and characterized as AR inhibitors with negligible (e.g., compound **1b)** or mild (e.g., compounds **2b,c**) efficacy and selectivity yet with significant antioxidant (AO) effect as an additional biological activity. The hexahydropyridoindole scaffold was excluded from further drug design since the marked antioxidant activity of the hexahydropyridoindoles was combined with only minor AR inhibition (e.g., compound **1b**), which was explained by space distortion of the hexahydropyridoindole structure not fitting properly the AR binding pocket. Tetrahydropyridoindole congeners with a planar tricyclic moiety appeared more promising route in designing efficient ARIs. Notably, elimination of the basicity center at *N*2 position, which prevented zwitterions formation, significantly improved AR inhibition as shown in the case of compound **2e**. 

Shifting the carboxymethyl pharmacophore from position 8 to position 2, yielded derivatives with markedly decreased inhibition efficacy, as exemplified by compound **2g**, therefore this route of drug designing was not followed further.

Structure optimization of the tetrahydropyridoindole scaffold by transferring the carboxymethyl pharmacophore from position 8 to position 5, yielded derivatives with markedly enhanced inhibition efficacy and selectivity, yet with abolished AO activity (e.g., compounds **3a**,**d**,**f**). In this series, the AR inhibition efficacy markedly increased with decreasing basicity of *N*2 nitrogen as documented by compound **3f.**

Thioxotriazine structural alternatives yielded highly efficient AR inhibitors (e.g., compound **5a**) with high selectivity and reasonable AO activity. In further structure optimization efforts, isosteric replacement of sulfur in compound **5a** with oxygen provided compound **7a** with increased AR inhibition efficacy and markedly improved selectivity, yet with diminished AO activity.

Interestingly, the structural features of the most efficient ARIs resulting from the above optimization procedure, namely compounds **2c**, **3f**, **5a,** and **7a** (IC_50_(ALR2) = 18,000; 13; 97; 42 nM, respectively) still match the strict criteria of the “rule of three“, which points to their excellent “lead-likeness” with prospects of further structure optimizations. Based on the experimental data available, summarized in Table 15, the lead-likeness score is favoring compounds **5a** (cemtirestat, CMTI) and **7a** (OTI) as promising scaffolds for further structure optimization. 

Four major routes of possible structural modifications of the leads with the aim to increase inhibition activity, to improve selectivity, bioavailability, and ADME properties, have been envisaged:

Replacement of the carboxymethyl pharmacophore by bioisosteric group of lower acidity, with the aim to improve bioavailability while binding affinity is preserved. 

Introduction of additional substituents with a variable H-bonding ability and lipophilicity into *N*2 position. Additional interactions with the unoccupied interactive pocket of ALR2 are expected to increase the selectivity as suggested by Hlavac et al. [80].

Aromatic substitutions by OH and/or OCH_3_ groups in variable positions relative to the indole nitrogen, with the aim to obtain bifunctional derivatives combining AR inhibition with antioxidant activity.

Syntheses of both symmetrical and asymmetrical disulfides of variable lipophilicity in the array of 5-carboxymethyl thioxotriazinoindoles. As a result, the disulfides may serve as prodrugs of efficient ARIs with an opportunity of targeted delivery into e.g., cancer cells endowed with high GSH. Therapeutic potential of the disulfide prodrugs in relation to several types of chronic inflammation-related cancer is expected (P5).

**Figure 18:**
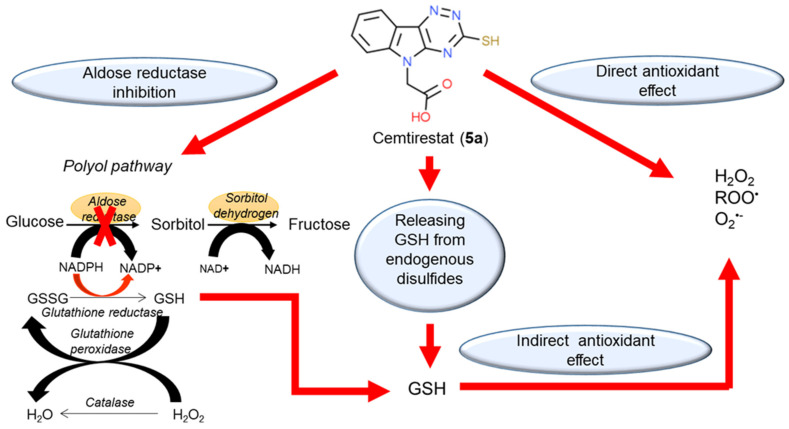
Multiple pharmacological activities of cemtirestat (**5a**). Reprinted with permission from [89]. Copyright (2020) Elsevier.

Among the novel derivatives, compound **5a** was patented (P3). At the present time, it is in the stage of complex preclinical evaluation under the name of cemtirestat [76,78,87,88,89,94,95,96,97,98,99,100]. Apparently, there are several advantages of cemtirestat over the clinically used epalrestat, namely lower molecular weight, better water solubility, higher AR inhibition activity recorded both at the level of isolated enzyme (Table 2 and Table 4) and at the organ level of isolated rat eye lenses (Table 13) and additional AO action (Table 10 and Table 11, Figure 15 and Figure 16). Recently, antioxidant activity of cemtirestat was corroborated by a study of Valachova et al. [96] reporting ability of the drug to protect hyaluronan against oxidatively induced degradation. In addition, protective effects of cemtirestat in neuron-like PC12 cells and BV2 rodent microglial cells exposed to toxic models of oxidative stress were recently documented [97,98]. Studies in vivo in rat models of diabetes revealed ability of cemtirestat (**5a**) to attenuate symptoms of peripheral neuropathy with high significance [89,95,100]. Moreover, our very recent study [89] proved that the direct ROS scavenging activity of cemtirestat (**5a**) is complemented by its ability to restore thiol-disulfide homeostasis by releasing free GSH from the pool of endogenously bound disulfides. In addition, ceasing aldose reductase activity by cemtirestat spares NADPH, which is needed for recycling of GSH by glutathione reductase. Considering the above-mentioned complementary activities (Figure 18), cemtirestat (**5a**) represents a practical example of a therapeutic strategy against chronic complications in diabetes based on multiple pharmacological activities. Yet, in-depth studies of the indicated molecular mechanisms and their mutual interactions are still needed.

## 5. Patents

1. Štefek, M.; Šnirc, V.; Demopoulos, V.; Djoubissie, P.; Račková, L.; Májeková, M.; Karasu, C. Carboxymethylated pyridoindoles as pharmaceutical agents, 8.6.2009, Slovak Patent No. 28695.

2. Štolc, S.; Považanec, F.; Bauer, V.; Májeková, M.; Wilcox, A.; Šnirc, V.; Račková, L.; Sotníková, R.; Štefek, M.; Gáspárová-Kvaltínová, Z.; Gajdošíková, A.; Mihálová, D.; Alföldi, J. Pyridoindole derivatives with antioxidant properties, preparation and therapeutic use, 7.12.2010, Slovak Patent No. 287506.

3. Štefek, M.; Miláčková; Diez-Dacal, B.; Pérez-Sala, D.; Šoltésová Prnová, M. Use of 5-carboxymethyl-3-mercapto-1,2,4-triazino-[5,6-b]indoles and their pharmaceutical composition, 3.4.2015, WO2015057175; 3.10.2017 Slovak Patent No. 288508.

4. Štefek, M.; Ballekova, J.; Šoltesová-Prnová, M.; Májeková, M. Use of 5-Carboxymetyl-1,2,3,4-tetrahydro-1H-pyrido[4,3-b] indoles and their pharmaceutical composition, 7.1.2020, Slovak Patent No. 288725.

5. Štefek, M.; Kováčiková, L.; Šoltésová Prnová, M.; Addová, G.; Boháč, A. Cemtirestat disulfide prodrug of aldose reductase inhibitor, preparation, pharmaceutical composition and therapeutic use, 14.12.2020, Slovak Patent Application No. 50074-2020.

## Figures and Tables

**Table 1:**
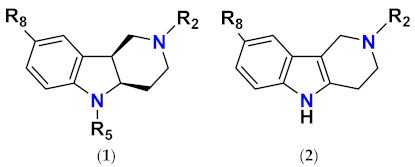
Inhibition of rat lens ALR2 and rat kidney ALR1.

Compound	Substituent			ALR2	ALR1	SF ^a^	Reference
	R2	R5	R8	IC_50_ (µM)/I (%,100 µM)	IC_50_ (µM)		
**1a** (STB)	-CH_3_	-H	-CH_3_	>100/1	n.d.	-	[63]
**1b**	-CH_3_	-H	-CH_2_COOH	>100/13	n.d.	-	[63]
**1c**	-CH_2_Ph	-H	-CH_2_COOH	>100/19	>3.000	-	[63]
**1d**	-COOEt	-H	-OCH_3_	>100/1	n.d.	-	[67]
**1e**	-CH_3_	-COCH_3_	-CH_3_	>100/1	n.d.	-	[67]
**2a**	-CH_3_	-H	-CH_3_	>100/1	n.d.	-	[63]
**2b**	-CH_3_	-H	-CH_2_COOH	54/n.d.	3.081	57	[63]
**2c**	-CH_2_Ph	-H	-CH_2_COOH	18/n.d.	328	18	[63]
**2d**	-CH_2_CH_2_Ph	-H	-CH_2_COOH	16/n.d.	603	38	[63]
**2e**	-COOEt	-H	-CH_2_COOH	4/n.d.	n.d.	-	[68]
**2f**	-CH_2_COOH	-H	-H	>100/38	n.d.	-	[67]
**2g**	-CH_2_COOH	-H	-CH_3_	>100/22	n.d.	-	[67]
**2h**	-CH_2_COOH	-H	-OCH_3_	>100/14	n.d.	-	[67]

^a^ SF means a selectivity factor defined as IC_50_(ALR1)/IC_50_(ALR2). The values of SF may differ slightly from those shown in the original publication due to rounding of the IC_50_ values.

**Table 2:**
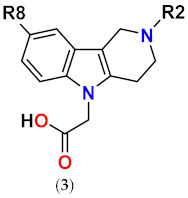
Inhibition of rat lens ALR2 and rat kidney ALR1.

Compound	Substituent		ALR2	ALR1	SF ^a^	Reference
	R2	R8	IC_50_ (nM)	IC_50_ (µM)		
**3a**	-*i*Pr	-H	34,250	>100	>3	[71]
**3b**	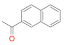	-F	141	44	312	[71]
**3c**	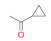	-H	57	8	140	[71]
**3d**	-COCH_3_	-H	21	22	1048	[71]
**3e**	-COOEt	-COOH	13	5	381	[71]
**3f**	-COOEt	-OCH_3_	13	10	792	[71]
epalrestat			227	-	-	[71]
valproic acid			-	56	-	[71]

^a^ SF means a selectivity factor defined as IC_50_(ALR1)/IC_50_(ALR2). The values of SF may differ slightly from those shown in the original publication due to rounding of the IC_50_ values.

**Table 3:**
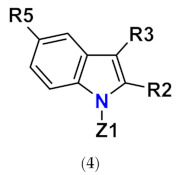
Inhibition of rat lens ALR2 and rat kidney ALR1.

Compound	Substituent				ALR2	ALR1	SF ^a^	Reference
	Z1	R2	R3	R5	IC_50_ (µM)/I (%,100 µM)	IC_50_ (µM)		
**4a**	-CH_2_COOH	-H	-H	-H	7/n.d.	80/n.d.	11	[76]
**4b**	-CH_2_CH_2_COOH	-H	-H	-H	100/54	>100/12	>1	[76]
**4c**	-H	-H	-CH_2_COOH	-H	>100/38 ^b^	n.d./n.d.	-	[75]
**4d**	-H	-H	-CH_2_COOH	-OCH_3_	>100/11 ^b^	n.d./n.d.	-	[75]
**4e**	-CH_2_COOH	-H	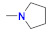	-H	46/n.d.	>100/20	>2	[76]
**4f**	-CH_2_COOH	-CH_3_	-CH_2_N(CH_3_)_2_	-H	42/n.d.	>100/22	>2	[76]
**4g**	-CH_2_COOH	-H	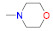	-H	35/n.d.	>100/32	>2	[76]
**4h**	-CH_2_COOH	-H	-CH_2_N(Et)_2_	-H	30/n.d.	>100/38	>3	[76]
**4i**	-CH_2_CH_2_COOH	-H	-CH_2_N(Et)_2_	-H	n.d./20	>100/7	-	[76]
**4j**	-CH_2_COOH	-H	-CO^t^Bu	-H	14/n.d.	68/n.d.	5	[76]
**4k**	-CH_2_COOH	-CH_3_	-CHO	-H	0.3/n.d.	12/n.d.	40	[71]
**4l**	-CH_2_COOH	-CH_3_	-COCH_3_	-H	0.2/n.d.	11/n.d.	55	[71]
**4m**	-CH_2_COOH	-H	-H	-OCH_2_Ph	0.7/n.d.	5/n.d.	7	[77]

^a^ SF means a selectivity factor defined as IC_50_(ALR1)/IC_50_(ALR2). ^b^ Rabbit lens ALR2. The values of SF may differ slightly from those shown in the original publication due to rounding of the IC_50_ values.

**Table 4:**
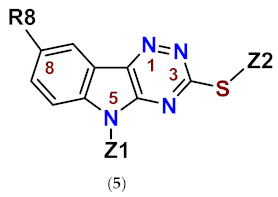
Inhibition of rat lens ALR2 and rat kidney ALR1.

Compound	Substituent			ALR2	ALR1	SF ^a^	Reference
	Z1	Z2	R8	IC_50_ (nM)/I (%,100 µM)	IC_50_ (µM)/I (%,100 µM)		
**5a** (CMTI)	-CH_2_COOH	-H	-H	97/n.d.	41/n.d.	422	[76]
**5b**	-H	-H	-H	>100/4	n.d./n.d.	-	[76]
**5c**	-H	-CH_2_COOH	-H	>100/3	n.d./n.d.	-	[76]
**5d**	-CH_2_COOH	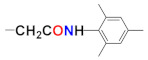	-H	4060/n.d.	n.d./24	-	[76]
**5e**	-CH_2_COOH	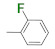	-H	2240/n.d.	n.d./1.2	-	[76]
**5f**	-CH_2_COOH	-CH(CH_3_)_2_	-H	2050/n.d.	14/n.d.	7	[76]
**5g**	-CH_2_COOH	-CH_2_CONHCH_2_CH_2_CH_3_	-CH_3_	330/n.d.	20/n.d.	61	[76]

^a^ SF means a selectivity factor defined as IC_50_(ALR1)/IC_50_(ALR2). The values of SF may differ slightly from those shown in the original publication due to rounding of the IC_50_ values.

**Table 5:**
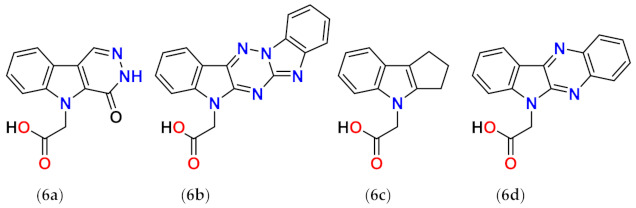
Inhibition of rat lens ALR2 and rat kidney ALR1.

Compound	ALR2	ALR1	SF ^a^	Reference
	IC_50_ (µM)	IC_50_ (µM)/I (%,100 µM)		
**6a**	12	100/49	8	[76]
**6b**	10	n.d./n.d.	-	[76]
**6c**	3	5/n.d.	2	[76]
**6d**	3	25/n.d.	8	[76]

^a^ SF means a selectivity factor defined as IC_50_(ALR1)/IC_50_(ALR2). The values of SF may differ slightly from those shown in the original publication due to rounding of the IC_50_ values.

**Table 6:**
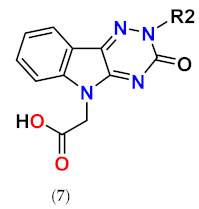
Inhibition of rat lens ALR2 and rat kidney ALR1.

Compound	Substituent	ALR2	ALR1	SF ^a^	Reference
	R2	IC_50_ (nM)	IC_50_ (µM)/I (%,100 µM)		
**7a** (OTI)	-H	42	>100/24	>2381	[79]
**7b**	-CH_2_COOH	120	20/n.d.	166	[79]
**7c**	-CH_2_Ph	434	>100/38	>230	[79]
**7d**	-CH_2_OCH_3_	85	>100/13	>1177	[79]

^a^ SF means a selectivity factor defined as IC_50_(ALR1)/IC_50_(ALR2). The values of SF may differ slightly from those shown in the original publication due to rounding of the IC_50_ values.

**Table 7:**
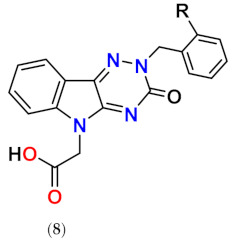
Inhibition of rat lens ALR2 and rat kidney ALR1.

Compound	Substituent	ALR2	ALR1	SF ^a^	Reference
	R	IC_50_ (nM)	IC_50_ (µM)/I (%,100 µM)		
**7c**	-H	434	>100/38	>230	[79]
**8a**	-CN	76	100/51	1316	[80]
**8b**	-CONH_2_	236	100/50	424	[80]
**8c**	-COOH	139	59/62	424	[80]
**8d**	-CH_2_OH	244	>100/33	>410	[80]

^a^ SF means a selectivity factor defined as IC_50_(ALR1)/IC_50_(ALR2). The values of SF may differ slightly from those shown in the original publication due to rounding of the IC_50_ values.

**Table 8:**
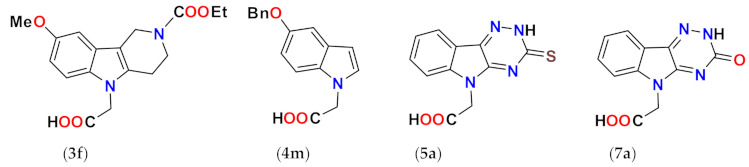
Inhibition of human recombinant enzymes AKR1B1 and AKR1B10 by the most promising ARIs in comparison with the rat ALR2.

Compound	IC_50_ (nM)/SF		Reference
	rat-ALR2	Human-AKR1B1	
**3f**	13/792 ^a^	84/112 ^b^	[71]
**4m**	700/7 ^a^	5400/n.d. ^b^	[77]
**5a** (CMTI)	100/422 ^a^	57/375 ^b^	[76]
**7a** (OTI)	42/>2 381 ^a^	66/852 ^b^	[79]

^a^ SF means a selectivity factor defined as IC_50_(ALR1)/IC_50_(ALR2); ^b^ SF defined as IC_50_(AKR1B10)/IC_50_(AKR1B1).

**Table 9:** Antiradical activities in a DPPH test ^a^.

Compound	Absorbance Decrease (-ΔA/5 min)	Reference
**1a** (STB)	0.239	[63,83,86]
**1b**	0.132	[63]
**1c**	0.187	[63]
**1d**	0.514	[86]
**1e**	<0.016	[83]
**2a**	0.033	[63,83]
**2b**	0.031	[63]
**2c**	0.030	[63]
**2d**	0.031	[63]
trolox	0.503	[83,86]

^a^ The ethanolic solution of DPPH radical (50 μM) was incubated in the presence of the compound tested (50 μM). Absorbance decrease at 518 nm during the first 5 min interval was determined.

**Table 10:** Antiradical activities in a DPPH test ^a.^

Compound	Absorbance Decrease (-ΔA/30 min)	Reference
**4a**	0.001	[75]
**4c**	0.069	[75]
**4d**	0.089	[75]
**5a** (CMTI)	0.219	[87]
**5f**	0.009	[87]
**7a** (OTI)	0.057	[79]
**7d**	0.042	[79]
melatonin	0.022	[79]

^a^ The ethanolic solution of DPPH radical (50 μM) was incubated in the presence of the compound tested (200 μM). Absorbance decrease at 518 nm during the first 30 min interval was determined.

**Table 11:** Inhibition of AAPH-induced peroxidation of DOPC liposomes by compounds **1a,c**; **2a,c;** and **5a,f** in comparison with the standard trolox and melatonin.

Compound	IC_50_ (µM)	Reference
**1a** (STB)	25.3	[63]
**1c**	75.6	[63]
**2a**	72.7	[63]
**2c**	168.1	[63]
**5a** (CMTI)	121.9	[89]
**5f**	>250	[68]
trolox	86.0	[68]
melatonin	131.1	[89]

DOPC liposomes (0.8 mM) were incubated in the presence of AAPH (10 mM) in phosphate buffer (pH 7.4, 20 mM) at 50 °C for 80 min.

**Table 12:** Effects of compound **1a** (stobadine) and compound **1d** on AAPH- or *t*-BuOOH-induced hemolysis of isolated rat erythrocytes.

Compound	Lag Time (min) ^a^	
	AAPH	*t*-BuOOH
Control	80	84
**1a** (STB), 10 µM	126	157
**1d**, 10 µM	90	257

^a^ Erythrocyte suspensions (1.5%) were incubated with 30 mM AAPH or 250 µM *t*-BuOOH. in the absence (Control) or the presence of the indicated compounds [86].

**Table 13:** Effect of the aldose reductase inhibitors **2c**, **3d, 3f, 4m, 5a,** and **7a** (IC_50_(ALR2): 18,000; 21; 13; 700; 97 and 42 nM, respectively) on sorbitol accumulation in the isolated rat eye lenses incubated ex vivo with high glucose in comparison with standard epalrestat_._

Compound	IC_50_ (µM)/I (%,10 µM)	Reference
**2c**	~100/5	[93]
**3d**	~10/52	[71]
**3f**	~10/53	[71]
**4m**	~100/28	[77]
**5a** (CMTI)	~100/38	[76]
**7a** (OTI)	~10/51	[79]
epalrestat	>50/12	[71,77]

Glucose, 50 mM; time of incubation, 3 h; 37 °C.

**Table 14:** Accumulation of sorbitol in the sciatic nerve of the rats under conditions of STZ-induced experimental diabetes. Effect of the compounds **2c, 3f** and **5a** (IC_50_(ALR2): 18,000, 13, 97 nM, respectively) ^a^.

	Sorbitol in the Sciatic Nerve, I (%) ^b^		
	2c [93]	3f [71]	5a (CMTI) [94]
Drug treated diabetic rats	25	19	20

^a^ The drugs were administered intragastrically for five consecutive days according to the following dosage schedule: 25 mg/kg twice daily (8:30 and 15:30) for the first four days and 25 mg/kg on the fifth day three hours before sacrificing the animals. ^b^ Percentage of the inhibition relative to the control untreated rats.

**Table 15:** Lead likeness score of compounds **2c**, **3f**, **5a** and **7a** (IC_50_(ALR2): 18,000; 13; 97 and 42 nM, respectively).

**Property/Compound**	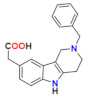 **2c**	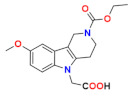 **3f**	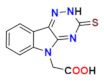 **5a (CMTI)**	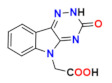 **7a (OTI)**
Rule of 3				
AR inhibition in vitro				
Inhibition of sorbitol accumulation in the eye lens ex vivo				
Inhibition of sorbitol accumulation in the sciatic nerve in vivo				n.d.
DPPH scavenging				
Inhibition of AAPH-induced peroxidation of DOPC liposomes				

□—excellent; □—medium; □—poor; n.d.—not determined.
